# Prevalence of Metabolic Syndrome and Its Associated Factors among Vegetarians in Malaysia

**DOI:** 10.3390/ijerph15092031

**Published:** 2018-09-17

**Authors:** Yuan Kei Ching, Yit Siew Chin, Mahenderan Appukutty, Wan Ying Gan, Vasudevan Ramanchadran, Yoke Mun Chan

**Affiliations:** 1 Department of Nutrition and Dietetics, Faculty of Medicine and Health Sciences, Universiti Putra Malaysia, 43400 Seri Kembangan, Selangor, Malaysia; ykching2010@gmail.com (Y.K.C.); wanying@upm.edu.my (W.Y.G.); cym@upm.edu.my (Y.M.C.); 2 Research Centre of Excellence, Nutrition and Non-Communicable Diseases, Universiti Putra Malaysia, 43400 Seri Kembangan, Selangor, Malaysia; 3 Programme of Sports Science, Faculty of Sports Science and Recreation, Universiti Teknologi MARA, 40450 Shah Alam, Selangor, Malaysia; mahen@salam.uitm.edu.my; 4 Malaysia Research Institute on Ageing (MyAgeing), Universiti Putra Malaysia, 43400 Seri Kembangan, Selangor, Malaysia; vasudevan@upm.edu.my

**Keywords:** metabolic syndrome, vegetarian, vegetarianism practises, overweight, lifestyle behaviours

## Abstract

The prevalence and factors associated with metabolic syndrome (MetS) remain unknown in Malaysian vegetarians. This cross-sectional study aimed to determine the prevalence of MetS among vegetarians in Kuala Lumpur and Selangor and its associated factors. The data on socio-demographic characteristics, vegetarianism practises, lifestyle behaviours, body weight, height, waist circumference (WC), systolic blood pressure (SBP), diastolic blood pressure (DBP), fasting blood glucose (FBG), and blood lipid profiles were collected from 273 vegetarians. A majority of the respondents were lacto-ovo vegetarians (44.0%), females (64.8%) and Chinese (54.9%). The prevalence of MetS was 24.2%. High BP (48.7%) and high WC (43.6%) were the most common MetS components. Females had lower WC, SBP, DBP, FBG, TG and higher HDL-c (*p* < 0.05) as compared to males. Multiple logistic regression analysis showed that being overweight and obese (Odds Ratio (OR) = 7.74, 95% Confidence Interval (CI): 4.04–14.82) was the main risk factor of MetS after being adjusted for sex and age. This study found that one in four vegetarians had MetS. An intervention programme should be developed to reduce Body Mass Index (BMI) among vegetarians, especially among those who are found to be overweight and obese.

## 1. Introduction

Metabolic syndrome (MetS) is a constellation of metabolic abnormalities including abdominal obesity, elevated blood pressure (BP) [systolic blood pressure (SBP) or diastolic blood pressure (SBP)], elevated fasting blood glucose (FBG), and dyslipidaemia [elevated triglyceride levels (TG) and low high-density lipoprotein cholesterol levels (HDL-c)] that promotes the development of cardiovascular disease (CVD) and type 2 diabetes mellitus (T2DM) [1]. Several criteria for diagnosis of MetS were introduced by different organisations between 1998 and 2009, including the World Health Organisation (WHO) in 1998 [2], the National Cholesterol Education Program Expert Panel on Detection, Evaluation, and Treatment of High Blood Cholesterol in Adults (Adult Treatment Panel III) (NCEP-ATP III) in 2002 [3], the American Heart Association/National Heart, Lung, and Blood Institute (AHA/NHLBI) [1] and International Diabetes Federation (IDF) in 2005 [4]. The Joint Interim Statement (JIS) is the latest criterion that was introduced by IDF; AHA/NHLBI; American Heart Association (AHA); World Heart Federation (WHF); International Atherosclerosis Society (IAS); and International Association for the Study of Obesity (IASO) in 2009 [5] due to the debates and controversies on different MetS definition over the years. According to the JIS criteria, MetS is characterised by the presence of at least three of the following components: abdominal obesity, as defined according to the Asian populations with large waist circumference (≥90.0 cm for men and ≥80.0 cm for women), low HDL-c (<1.0 mmol/L for men and <1.3 mmol/L for women), high TG (≥1.7 mmol/L), high FBG (≥5.6 mmol/L), and high BP (SBP ≥ 130 mmHg or DBP ≥ 85 mmHg) [5].

MetS has become a public health concern due to the increased number of non-communicable diseases (NCDs) around the world over these past few decades [6,7,8]. The prevalence of MetS varies according to different definitions and populations [6,8,9]. For instance, in the United States (U.S.), the prevalence of MetS increased from 32.9% in 2004 to 34.7% in 2015 according to criteria by NCEP-ATP III [6]. In Korea, the prevalence of MetS increased from 24.9% in 1998 to 31.3% in 2007 according to criteria by NCEP-ATP III [9]. In Malaysia, the latest prevalence of MetS as defined by JIS was 42.5% in 2013 [8]. While the prevalence of MetS was observed in many countries among the general population [7,8,9,10], several studies have reported that vegetarians are associated with a lower risk of MetS as compared to the general population [11,12,13]. For example, the prevalence of MetS among U.S. vegetarians (25.2%) were lower than U.S. non-vegetarians (39.7%) [11]. Similarly, the prevalence of MetS among Taiwanese female vegetarians were lower than non-vegetarians according to criteria by NCEP ATP-III (13.3% vs. 20.0%), and IDF (6.7% vs. 12.7%), respectively [12]. Likewise, postmenopausal vegetarians had a lower risk of MetS (33.9%) than non-vegetarians (47.9%) in Korea [13]. The existing studies, which compared the prevalence of MetS between vegetarians and non-vegetarians found that vegetarians had lower prevalence of MetS than non-vegetarians [11,12,13,14]. Despite the lower prevalence of MetS in vegetarians than non-vegetarians, the MetS issues in vegetarians (6.7–33.9%) [11,12,13,14] should not be neglected. To date, there is no published study on MetS among Malaysian vegetarians. To the best of our knowledge, the existing studies on Malaysian vegetarians focussed on reasons for being a vegetarian, their nutrient intakes, and CVD risk profiles [15,16,17]. There are limited studies reporting on the associations between vegetarian lifestyle and MetS, when vegetarians are often viewed as the “healthier group” as compared to non-vegetarians [18]. Previous studies have reported that lifestyle factors were associated with MetS, and these studies were conducted among the general population [7,8,19]. Therefore, the current study aimed to determine the lifestyle factors of vegetarians, and its associated factors with MetS, which would give us an overall picture of the MetS issue in a sample of Malaysian vegetarians.

## 2. Materials and Methods

### 2.1. Study Design and Study Population

The present study was approved by the Ethics Committee for Research involving Human Subjects, Universiti Putra Malaysia (JKEUPM) [Reference number: FPSK (FR16) P023]. Power calculation was used to determine the sample size required. Data collection was conducted among Malaysian vegetarians at selected community centres located in Kuala Lumpur and Selangor. Members of the community centres were mainly vegetarians. Prior to data collection, lists of community centres were obtained from its headquarter (HQ). A total of nine community centres were randomly selected from 31 community centres. All members of the selected community centres were invited to participate in the study. A study information sheet explaining the purpose of the study was distributed to the respondents. Respondents were screened for the study criteria, namely adults aged above 18 years old, practising vegetarianism for more than 2 years, not pregnant or lactating, and not taking medications in controlling dyslipidaemia, diabetes, or hypertension. Signed consents were obtained from all respondents who agreed to participate in the study. Respondents who fulfilled the study criteria were then requested to fast overnight for at least 8 h, in preparation for blood withdrawal on the morning of the data collection day. Respondents were asked to complete a set of self-administered questionnaires. Their body weight, height, WC, and BP were measured by the researchers.

### 2.2. Socio-Demographic Characteristics

In this section, socio-demographic characteristics such as sex, age, marital status, monthly household income, and education level were self-reported by the respondents. Total household income (in Ringgit Malaysia, RM 1.00 = US Dollar 0.26) was categorised into low (<RM 2300 or USD 598), medium (RM 2300–5599 or USD 598–1456) and high (≥RM 5600 or USD 1457) based on the Malaysian total household income stated in the 10th Malaysia Plan [20].

### 2.3. Vegetarianism Practices

Respondents were asked to indicate their current vegetarianism practice: (i) vegans who only consume plant-based foods and no meat, fish, poultry, dairy products, and eggs; (ii) lacto-vegetarians who consume dairy products but no eggs, fish, meat, and poultry; (iii) ovo-vegetarians who consume eggs but no dairy products, fish, meat, and poultry; and (iv) lacto-ovo-vegetarians who consume dairy products and eggs, but no fish, meat, and poultry [21]. Respondents self-reported the number of years they have been practising vegetarianism. In addition, they were asked about the reasons for adopting vegetarianism.

### 2.4. Lifestyle Behaviours

Lifestyle behaviours of the respondents such as alcohol consumption, smoking behaviour, and physical activity levels were assessed in the present study. Alcohol consumption was assessed through the adapted alcoholic questionnaire in the National Health and Nutrition Examination Survey (NHANES) Food Frequency Questionnaire (FFQ), and classified into two categories, namely alcohol users or non-alcohol users [22]. Smoking behaviour was assessed using a self-administered Global Adult Tobacco Survey (GATS) [23]. Current and past smoking behaviours of the respondents were assessed by three questions in GATS, and classified into three categories, namely smokers, past smokers and non-smokers [23]. Lastly, Global Physical Activity Questionnaire (GPAQ) was used to assess the physical activity level of the respondents for the past week [24]. GPAQ consisted of three domains including activities at work, travel to and from places and recreational activities, whereby different types of activities were assigned to the designated METs values [24]. The MET-min week was calculated as minutes of activity/day × days per week × MET level. High physical activity level is defined as respondents having 7 or more days of any combination of walking, moderate or vigorous-intensity activities in achieving a minimum of at least 3000 MET-min per week, while moderate physical activity level is referred to those who had 5 or more days of any combination of walking, moderate-, or vigorous intensity activities achieving a minimum of at least 600 MET minutes/week and a maximum of 2999 MET minutes/week Respondent who does not meet the high or moderate physical activity level criteria were classified as having low physical activity level [24].

### 2.5. Anthropometric Assessments

Anthropometric measurements were measured according to the International Standards for Anthropometric Assessment (ISAK) method. Body weight of the respondent was measured using TANITA Digital Weight Scale HD306 (TANITA Corporation, Arlington Heights, IL, USA) to the nearest 0.1 kg. Meanwhile, height of the respondent was measured using a portable SECA213 portable stadiometer (SECA, Hamburg, Germany) to the nearest 0.1 cm. Body mass index (BMI) was calculated as kg/m^2^, and respondent’s BMI was categorised into underweight, normal, overweight and obesity [25]. WC of the respondent was measured using a Lufkin tape W606PM (Lufkin, Lufkin, TX, USA) to the nearest 0.1 cm. WC cut-off points of ≥90.0 cm for men and ≥80.0 cm for women were considered as at-risk of having abdominal obesity [26]. WC was one of the components to define MetS [5].

### 2.6. Blood Pressure Assessment

Blood pressure (SBP and DBP) of the respondent was measured in mmHg using an Omron automatic blood pressure monitor (HEM-7121, Omron Corporation, Kyoto, Japan). Respondents were requested to rest and sit on a chair before the measurement. According to the WHO/International Society of Hypertension, respondents were considered at risk of high-normal blood pressure if SBP ≥ 130 mmHg or DBP ≥ 85 mmHg [27]. High BP was one of the components to define MetS [5].

### 2.7. Biochemical Assessments

A total of 5 mL of overnight venous fasting blood sample was drawn by a registered nurse in the morning time of the data collection day. Blood samples were kept in the icebox and sent to the laboratory on the same day of data collection for further analysis. Olympus Au analyser (AU640, Beckman Olympus, Tokyo, Japan) was used to determine the blood glucose level and blood lipid profiles (HDL-c and TG). Respondents were considered at risk of low HDL-c if HDL-c < 1.0 mmol/L for men and <1.3 mmol/L for women, and high TG if ≥1.7 mmol/L according to the NCEP-ATP III [28]. Low HDL-c and high TG were the two components to define the MetS [5]. Meanwhile, respondents with FBG ≥ 5.6 mmol/L were classified as having abnormal blood glucose level as defined by American Diabetic Association in 2003 [29]. High blood glucose was one of the components to define MetS [5].

### 2.8. Statistical Analysis

Data were analysed using IBM SPSS Statistics 24.0 (SPSS Inc., Chicago, IL, USA) software. Continuous variables were tested for normality, and variables that fall within skewness of ±2 were considered normally distributed [30]. The differences in the variables between respondents with MetS and without MetS were tested using independent samples *t-*test for normally distributed data and Mann–Whitney test for non-normally distributed data. Chi-square analysis was used to determine the association of categorical variables with MetS status. Multiple logistic regression was used to determine the risk factors of MetS after being adjusted for sex and age. The odds ratios (ORs) and 95% confidence intervals (CIs) were determined. The level of significance was set at *p* < 0.05. 

## 3. Results

### 3.1. General Characteristics

Socio-demographic characteristics, vegetarianism practices, and body compositions of the respondents are presented in Table 1. The present study consisted of 44.0% lacto-ovo vegetarians, followed by lacto-vegetarians (31.5%), vegans (19.0%) and ovo-vegetarians (5.5%). They had practised vegetarianism for an average of 14.2 ± 9.6 years. No significant difference was found in the number of years of practising vegetarianism between males (14.7 ± 9.5 years) and females (13.9 ± 9.7 years) (*p* > 0.05). The majority of the respondents practised vegetarianism due to religious belief (75.8%). One-third of the respondents (36.3%) had medium total household income (RM 2300–5599). About half (54.9%) of the respondents had normal BMI, 27.5% of the them were overweight, while 8.1% of the them were obese. With regards to the lifestyle behaviours, most of the respondents were non-smokers (95.2%), non-alcohol drinkers (91.9%), and had low PA level (46.1%) (Table 1).

### 3.2. Prevalence of MetS and Distribution of MetS Components

Table 2 shows the metabolic risks profile of the respondents. Based on JIS, the overall prevalence of MetS was 24.2%. The prevalence of MetS among males and females were 29.2% and 21.5%, respectively ($\chi^{2}$ = 2.01, *p* = 0.156). While 17.6% of the respondents did not fulfil any MetS criteria, Table 2 shows that a majority of them had at least one of the MetS components (mean number of MetS component: 1.7 ± 1.2). High BP (48.7%) and large WC (43.6%) were the two most common MetS components found among the vegetarians in the present study. Besides, females had lower WC, SBP, DBP, FBG, TG, and higher HDL-c (*p* < 0.05) as compared to males (Table 2). 

### 3.3. Factors Associated with MetS

Table 3 shows the factors associated with MetS among respondents. A high prevalence of MetS was observed among lacto-vegetarians (32.6%), followed by vegans (25.0%), lacto-ovo-vegetarians (19.2%), and ovo-vegetarians (13.3%). There was a statistically significant difference in the numbers of years of practising vegetarianism between respondents with MetS (17.9 ± 11.2 years) and those without MetS (13.0 ± 8.8 years) (*t* = −3.20, *p* = 0.0001). In addition, the age of respondents with MetS (51.1 ± 10.6 years) was statistically significantly higher than those without MetS (46.4 ± 13.6 years) (*t* = −2.89, *p* = 0.002). MetS was reported to be higher among respondents who were Indian (34.1%), married (30.0%), and overweight/obese (49.5%) as compared to their counterparts (*p* < 0.05). In contrast, there were no statistically significant differences in education levels, total household incomes, vegetarian categories, physical activity levels, alcohol consumption and smoking behaviour by MetS status (*p* > 0.05). Table 4 shows the multiple logistic regression analysis in the present study. The number of years of practising vegetarianism (OR = 1.03, 95% CI: 1.00–1.07), and being overweight and obese (OR = 7.54, 95% CI: 3.97–14.30) were associated with MetS after the multiple logistic regression model was adjusted for the sex. A further adjustment of age showed that being overweight and obese (OR = 7.74, 95% CI: 4.04–14.82) remained as a significant risk factor of MetS among vegetarians in the present study. The multiple logistic regression model in the present study was able to classify 79.5% MetS cases correctly and explained 29.9% of the variances in MetS (Table 4).

## 4. Discussion

To the best of our knowledge, this is the first study that determined the prevalence of MetS among vegetarians in Malaysia and its associated factors. In the present study, the overall prevalence of MetS among Malaysian vegetarians was 24.2%. The prevalence of MetS in Malaysian vegetarians was lower than the overall prevalence of MetS in the Malaysian general population (42.5%) [8]. Notably, the prevalence of MetS in Malaysian vegetarians was lower than the Korean postmenopausal vegetarians (33.9%) [13], and US vegetarians (25.2%) [11], but higher than the Taiwanese vegetarians (13.3%) [12]. The discrepancies in prevalence could be due to the different MetS criteria used by these studies [11,12,13,14,31]. The present study showed that high BP (48.7%) and large WC (43.6%) were found to be the most common MetS components among the vegetarians. In contrast, Chiang and colleagues [12] found that low HDL-c (35.8%) and high BP (32.0%) were the most prevalent MetS components in Taiwanese female vegetarians. The inconsistent findings could be due to the variations in MetS criteria and respondents’ characteristics as the latter study [12] involved only female vegetarians, whilst the present study consisted of both male and female vegetarians.

Even though a previous study reported that vegetarians had lower risk of obesity [32], 35.6% of the vegetarians in the present study were found to be overweight and obese. Based on National Health and Morbidity Survey 2015 (NHMS 2015) [33], the prevalence of overweight and obesity among the general population in Malaysia was 47.7%. In the present study, about half of the vegetarians (43.6%) were abdominally obese, which was comparable to the prevalence of abdominal obesity among the general population in Malaysia (48.6%), as reported in NHMS 2015 [33]. In addition, the prevalence of abdominal obesity among vegetarians in the present study was even higher than the world prevalence (13.0%) in 2014 [34]. These findings revealed that vegetarians who were overweight and obese were associated with a higher risk of MetS. Overweight and obesity problem indicates the problem of over-nutrition, whereby energy intake was in excess as compared to energy expenditure [35]. However, the present study was not able to report on the energy intake and energy expenditure of the vegetarians. Since the dietary intake of vegetarians is different from the general population, general dietary guidelines such as Malaysian Dietary Guideline [36] may not be applicable for vegetarians. Currently, some countries such as Japan [37] and North America [38] have developed vegetarian dietary guidelines, whilst Malaysia is currently developing a dietary guideline for vegetarians. These dietary guidelines can serve as a reference for Malaysian vegetarians in planning their daily diet. Further studies are needed to determine the factors that are associated with overweight and obesity in vegetarians, particularly on their energy intake and energy expenditure. The determination of these factors is important for the planning of future weight management interventions targeted for vegetarians. To date, there is no published weight management intervention focusing on vegetarians.

Despite the prevalence of MetS in males (29.2%) being higher than females (21.5), there was no significant difference between the sexes (*p* = 0.156). The present study found that WC, SBP, DBP, TG, and low-HDL-c were significantly different between males and females. Males had larger WC, higher TG, FBG and BP, but a lower HDL-c than females. The present findings were consistent with a past study conducted by Chee et al. [39] among Malaysian employees. The plausible mechanism to explain these results is that the endogenous hormones of females are less atherogenic and therefore has lesser effect on insulin resistance, providing the protective effects towards MetS components [40]. 

Lifestyle behaviours were not associated with MetS in Malaysian vegetarians. Unlike a previous study that reported a significant association between smoking behaviour and alcohol consumption and MetS among the general population [7,41], the present study did not observe any association of smoking behaviour and alcohol consumption with MetS. In the present study, a majority of the vegetarians were non-smokers (95.2%), and non-alcohol drinkers (91.9%). These lifestyle behaviours could be related to religious belief, as religious belief (75.8%) was the main reason for their current practise of vegetarianism in the present study. Religious belief teaches their followers to use alcohol moderately [42] and avoid unhealthy lifestyle such as smoking [43]. Further analysis showed that the number of years of practising vegetarianism and “practising vegetarianism is environmentally friendly” appeared significant in the bivariate analysis, but were not significant risk factors of MetS in the multiple logistic regression analysis. This suggests the potential influences of these two factors towards MetS among vegetarians. To the best of our knowledge, there is no reported association between the vegetarianism practices and MetS among vegetarians in previous studies. More studies are therefore needed to explore these influences on reasons for practising vegetarianism and its association with MetS among vegetarians.

Besides that, physical activity level of the vegetarians was not associated with MetS in the present study. This finding was consistent with a past study conducted in Nigerian adults [44], and the authors suggested that physical activity intervention alone may not be effective in reducing the risk of MetS. In contrast, several studies have depicted that physically inactive was a risk factor for MetS [19,45,46]. The protective effect of physical activity towards MetS can be explained by its ability to modulate the human blood lipid profile [47], improved body weight status [48], reduced BP [49] and improved insulin sensitivity [50]. The present study found that nearly half of the vegetarians (46.2%) were physically inactive, and only 18.7% of them had high physical activity level. In the present study, the GPAQ was used to assess the physical activity level of the vegetarians, which required them to recall their physical activities over the past seven days. The retrospective characteristic of GPAQ was largely dependent on the memory of the respondents. Thus, this may have introduced recall bias, which can affect the association between physical activity level and MetS.

Despite the prevalence of MetS has been reported [11,12,13,14], factors that contribute to the development of MetS in vegetarians remain unknown. The present study showed that vegetarians who were overweight and obese had higher odds for MetS compared to those who were underweight and with normal weight, and this observation remained significant after adjustments have been made for sex and age in the multiple logistic regression analysis. This result was in accordance with a few past studies [51,52] as they found that overweight and obese individuals had higher likelihoods of MetS than underweight and normal weight individuals. Carnethon and colleagues [53] found that the prevalence of MetS increased by 23.0% for every additional 4.5 kg weight gained, and they suggested that obesity is an easily observed and measurable risk factor for MetS. Inflammation may be the key factor linking between the obesity and MetS. This can be explained by the high amount of macrophages accumulation around the dead adipocytes in the inflamed adipose tissue [54]. The high amount of proinflammatory substance such as cytokine is released from the inflamed adipose tissue, which positively correlates with the development of MetS, atherosclerosis, and T2DM [55]. In other words, inflammation in adipose tissue may be a significant contributor to MetS, especially among those who are obese. Given the high prevalence of overweight and obesity in vegetarians in the present study, there is an urgent need to develop a weight management programme for this target population.

The strength of the present study is that it is the first vegetarian study which focuses on MetS and its associated factors, and this may serve as baseline data for future researchers. There are a few limitations need to be addressed and taken into consideration. Firstly, this study is a cross-sectional study, therefore it is unable to detect the causal effect of the studied factors towards the development of MetS. Longitudinal cohort studies are recommended to clarify the causal relationship between the studied factors with MetS in vegetarians. Secondly, the dietary intake of the vegetarians was not included in the present study. Future research should consider the role of dietary intake in the aetiology of MetS among vegetarians. BMI is a common and widely used indicator to assess body weight status in many studies, including the NHMS study in Malaysia [33] and other studies worldwide [53,56]. The widely used BMI allows comparison to be made with other studies that used similar indicator in assessing body weight status. However, the use of BMI as a measure of obesity measure may be another limitation in the present study, whereby an individual may be misinterpreted as overweight or obese due to his/her high muscle mass. With that, body composition analysis including fat mass and muscle mass could be an alternative in determining the association between overweight and obesity and MetS among vegetarians. Next, the odds ratio in multiple logistic regression analysis may over exaggerate the association between the risk factors and MetS [57]. As the present study is a cross-sectional study, odds ratio is therefore inappropriate to be interpreted as risk ratio [57], especially when the MetS is more than 10.0% among vegetarians. Besides, non-vegetarians were not included in the present study as our study aimed to determine the risk factors for MetS in vegetarians. Future study may compare the effects of practising vegetarianism on MetS and its associated factors between vegetarians and non-vegetarians in Malaysia.

## 5. Conclusions

Overall, the prevalence of Mets among vegetarians was 24.2%. While limited studies focused on the lifestyle of the vegetarians and its association with MetS, the present study determined the association between lifestyle factors and MetS among vegetarians. Although vegetarians were generally perceived as healthier group, nearly half of the vegetarians (43.6%) from the present study had abdominal obesity and 35.5% of the them were overweight and obese, which highlights the need to establish specific weight management programmes for this population. The present study emphasises the importance of monitoring body weight status of vegetarians, as overweight and obesity was identified as the main risk factor for MetS among vegetarians. Appropriate intervention programmes to reduce body weight and BMI should be established among vegetarians, especially for those vegetarians who were overweight and obese, to reduce their risk of MetS or its related components.

## Figures and Tables

**Table 1:** General characteristics of vegetarians by sex.

Variables	Male (*n* = 96)	Female (*n* = 177)	Total (*n* = 273)	$\chi^{2}/t$	*p* Value
^†^ **Vegetarian classifications**				5.82	0.121
Lacto-ovo-vegetarians	33 (34.4)	87 (49.1)	120 (44.0)		
Ovo-vegetarians	6 (6.3)	9 (5.1)	15 (5.5)		
Lacto-vegetarians	37 (38.5)	49 (27.7)	86 (31.5)		
Vegans	20 (20.8)	32 (18.1)	52 (19.0)		
^⧧^ **Years of practising vegetarianism**					
Mean ± SD	14.7 ± 9.5	13.9 ± 9.7	14.2 ± 9.6	0.66	0.509
**Reasons for adopting vegetarianism**					
**Animal welfare**				0.01	0.923
Yes	38 (39.6)	69 (39.0)	107 (39.2)		
No	58 (60.4)	108 (61.0)	166 (60.8)		
**Health concern**				0.12	0.73
Yes	52 (54.2)	92 (52.0)	144 (52.7)		
No	44 (45.8)	85 (48.0)	129 (47.3)		
**Religious belief**				1.55	0.21
Yes	77 (80.2)	130 (73.4)	207 (75.8)		
No	19 (19.8)	47 (26.6)	66 (24.2)		
**Environmentally friendly**				0.99	0.32
Yes	39 (40.6)	83 (46.9)	122 (44.7)		
No	57 (59.4)	94 (53.1)	151 (55.3)		
**Family influence**				7.07	0.008 *
Yes	18 (18.8)	14 (7.9)	32 (11.7)		
No	78 (81.2)	163 (92.1)	241 (88.3)		
^⏀^ **Others**				-	0.427
Yes	1 (1.0)	6 (3.4)	7 (2.6)		
No	95 (99.0)	171 (96.6)	266 (97.4)		
^⧧^ **Age (years)**					
Mean ± SD	46.0 ± 14.5	48.4 ± 12.3	47.5 ± 13.1	−1.44	0.151
^†^ **Ethnicity**				7.49	0.006 *
Chinese	42 (43.8)	108 (61.0)	150 (54.9)		
Indians	54 (56.2)	69 (39.0)	123 (45.1)		
^†^ **Marital status**				5.49	0.064
Single	33 (34.4)	47 (26.6)	80 (29.3)		
Married	57 (59.4)	103 (58.2)	160 (58.6)		
Divorced/Widowed	6 (6.2)	27 (15.2)	33 (12.1)		
^†,^^⫮^ **Education**				4.49	0.106
Primary education	17 (17.7)	24 (13.6)	41 (15.0)		
Secondary education	43 (44.8)	103 (58.2)	146 (53.5)		
Tertiary education	36 (37.5)	50 (28.2)	86 (31.5)		
^†^ **Total household income**				0.83	0.661
<RM 2300	30 (31.3)	62 (35.0)	92 (33.7)		
RM 2300–5599	34 (35.4)	65 (36.7)	99 (36.3)		
≥RM 5600	32 (33.3)	50 (28.3)	82 (30.0)		
^⧧^ **Body weight (kg)**					
Mean ± SD	71.7 ± 12.5	57.3 ± 10.8	62.3 ± 13.4	9.92	0.0001 *
^⧧^ **Height (cm)**					
Mean ± SD	170.2 ± 6.8	157.3 ± 6.2	161.8 ± 8.9	15.84	0.0001 *
^†,^^⧧^ **BMI (kg/m^2^)**					
Mean ± SD	24.8 ± 4.4	23.10 ± 3.9	23.7 ± 4.1	3.37	0.001 *
Underweight	6 (6.3)	20 (11.3)	26 (9.5)	5.67	0.129
Normal	50 (52.1)	100 (56.5)	150 (54.9)		
Overweight	28 (29.1)	47 (26.6)	75 (27.5)		
Obesity	12 (12.5)	10(5.6)	22 (8.1)		
^†^ **Alcohol consumption**				0.35	0.556
Yes	9 (9.4)	13 (7.3)	22 (8.1)		
No	87 (90.6)	164 (92.7)	251 (91.9)		
^⏀^ **Smoking behaviour**				-	0.002 *
Past smoker	10 (10.4)	3 (1.7)	13 (4.8)		
Non-smoker	86 (89.6)	174 (98.3)	260 (95.2)		
^†^ **Physical activity levels**				7.48	0.024 *
Low	37 (38.5)	89 (50.3)	126 (46.1)		
Moderate	33 (34.4)	63 (35.6)	96 (35.2)		
High	26 (27.1)	25 (14.1)	51 (18.7)		

Note: BMI: body mass index, RM: *Ringgit Malaysia*. ^†^ Variables are presented as n (%), and tested by Chi-square test with value reported in $\chi^{2}$ and *p*. ^⧧^ Variables are presented as Mean ± SD, and tested by Independent samples *t*-test with value reported in *t* and *p*. ^⫮^ Education level was merged into three categories to perform valid Chi-square test with value reported in $\chi^{2}$ and p. ^⏀^ Variable is presented as *n* (%), and reported with Fisher Exact test as more than 20% of the cells had expected count of less than 5. * Indicates a significant difference at *p* < 0.05 by Chi-square test, Fisher Exact test or Independent samples *t*-test.

**Table 2:** Metabolic risks profile of vegetarians by sex.

Variables	Male (*n* = 96)	Female (*n* = 177)	Total (*n* = 273)	*t* $/\chi^{2}/U$	*p* Value
^†^ **MetS**				2.01	0.156
Yes	28 (29.2)	38 (21.5)	66 (24.2)		
No	68 (70.8)	139 (78.5)	207 (75.8)		
^⧧^ **MetS components**					
Mean ± SD	1.8 ± 1.2	1.6 ± 1.2	1.7 ± 1.2	1.57	0.119
^†^ **Number of MetS components**					
0	11 (11.5)	37 (20.9)	48 (17.6)	5.44	0.364
1	36 (37.5)	58 (32.7)	94 (34.4)	-	-
2	21 (21.9)	44 (24.9)	65 (23.8)	-	-
3	16 (16.6)	21 (11.9)	37 (13.6)	-	-
4	11 (11.5)	15 (8.5)	26 (9.5)	-	-
5	1 (1.0)	2 (1.1)	3 (1.1)	-	-
^†,^^⧧^ **WC (cm)**					
Mean ± SD	89.5 ± 11.7	78.4 ± 10.1	82.3 ± 11.9	8.15	0.0001 *
Large	41 (42.7)	78 (44.1)	119 (43.6)	0.05	0.829
Normal	55 (57.3)	99 (55.9)	154 (56.4)	-	-
^†,^^⧧^ **SBP (mmHg)**					
Mean ± SD	131.8 ± 16.3	125.9 ± 19.2	127.9 ± 18.4	2.56	0.011 *
High	53 (55.2)	73 (41.2)	126 (46.2)	4.86	0.027 *
Normal	43 (44.8)	104 (58.8)	147 (53.8)	-	-
^†,^^⧧^ **DBP (mmHg)**					
Mean ± SD	79.1 ± 11.0	74.1 ± 10.2	75.9 ± 10.7	3.79	0.0001 *
High	30 (31.2)	27 (15.3)	57 (20.9)	9.64	0.002 *
Normal	66 (68.8)	150 (84.7)	216 (79.1)	-	-
^†^ **Blood pressure (SBP/DBP) (mmHg)**				8.11	0.004 *
High	58 (60.4)	75 (42.4)	133 (48.7)	-	-
Normal	38 (39.6)	102 (57.6)	140 (51.3)	-	-
^†,^^⏀^ **FBG (mmol/L)**					
Median (IQR)	4.9 (4.6–5.5)	4.7 (4.4–5.2)	4.8 (4.4–5.3)	6943.00	0.013 *
High	22 (22.9)	28 (15.8)	50 (18.3)	2.10	0.148
Normal	74 (77.1)	149 (84.2)	223 (81.7)	-	-
^⧧^ **TC (mmol/L)**					
Mean ± SD	5.1 ± 1.1	4.8 ± 0.9	4.9 ± 1.0	2.52	0.012 *
^†,^^⧧^ **HDL-c (mmol/L)**					
Mean ± SD	1.6 ± 0.2	1.3 ± 0.3	1.3 ± 0.3	−5.53	0.0001 *
Low	18 (18.8)	67 (37.9)	85 (31.1)	10.59	0.001 *
Normal	78 (81.2)	110 (62.1)	188 (68.9)	-	-
^⧧^ **LDL-c (mmol/L)**					
Mean ± SD	3.2 ± 1.1	2.9 ± 0.8	3.0 ± 0.9	2.30	0.022 *
^†,^^⏀^ **TG (mmol/L)**					
Median (IQR)	1.4 (0.9–2.1)	1.0 (0.7–1.4)	1.1 (0.8–1.6)	5898.50	0.0001 *
High	36 (37.5)	31 (17.5)	67 (24.5)	13.43	0.0001 *
Normal	60 (62.5)	146 (82.5)	206 (75.5)	-	-

Note: MetS, metabolic syndrome; WC, waist circumference; SBP, systolic blood pressure; DBP, diastolic blood pressure; FBG, fasting blood glucose; TC, total cholesterol; HDL-c, high-density lipoprotein cholesterol; LDL-c, low-density lipoprotein cholesterol; TG, triglyceride; SD, standard deviation; IQR, interquartile range. ^†^ Variables are presented as *n* (%), and tested by Chi-square test with value reported in $\chi^{2}$ and *p*. ^⧧^ Variables are presented as Mean ± SD, and tested by Independent samples *t-*test with value reported in *t* and *p*. ^⏀^ Variable is presented in Median (IQR), and was tested by Mann–Whitney test with value reported in *U* and *p*. * Indicates a significant difference at *p* < 0.05 by Chi-square test, Mann–Whitney test or Independent samples *t*-test.

**Table 3:** Factors associated with MetS.

Variables	With MetS (*n* = 66)	Without MetS (*n* = 207)	Total (*n* = 273)	$\chi^{2}/t$	*p* Value
^†^ **Vegetarian classifications**				5.92	0.116
Lacto-ovo-vegetarians	23 (19.2)	97 (80.8)	120 (44.0)		
Ovo-vegetarians	2 (13.3)	13 (86.7)	15 (5.5)		
Lacto-vegetarians	28 (32.6)	58 (67.4)	86 (31.5)		
Vegans	13 (25.0)	39 (75.0)	52 (19.0)		
^⧧^ **Years of practising vegetarianism**					
Mean ± SD	17.9 ± 11.2	13.0 ± 8.8	14.2 ± 9.6	−3.20	0.0001 *
^⏀^ **Reasons for adopting vegetarianism**					
**Animal welfare**				1.99	0.159
Yes	21 (19.6)	86 (80.4)	107 (39.2)		
No	45 (27.1)	121 (72.9)	166 (60.8)		
**Health concern**				0.26	0.61
Yes	33 (22.9)	111 (77.1)	144 (52.7)		
No	33 (25.6)	96 (74.4)	129 (47.3)		
**Religious belief**				0.95	0.33
Yes	53 (25.6)	154 (74.4)	207 (75.8)		
No	13 (19.7)	53 (80.3)	66 (24.2)		
**Environmentally friendly**				4.54	0.03 *
Yes	22 (18.0)	100 (82.0)	122 (44.7)		
No	44 (29.1)	107 (70.9)	151 (55.3)		
**Family influence**				0.11	0.75
Yes	7 (21.9)	25 (78.1)	32 (11.7)		
No	59 (24.5)	182 (75.5)	241 (88.3)		
^⏀^ **Others**					
Yes	3 (42.9)	4 (57.1)	7 (2.6)	-	0.365
No	63 (23.7)	203 (76.3)	266 (97.4)		
^⧧^ **Age (years)**					
Mean ± SD	51.1 ± 10.6	46.4 ± 13.6	47.5 ± 13.1	−2.89	0.004 *
^†^ **Sex**				2.01	0.156
Male	28 (29.2)	68 (70.8)	96 (35.2)		
Female	38 (21.5)	139 (78.5)	177 (64.8)		
^†^ **Ethnicity**				12.14	0.0001 *
Chinese	24 (16.0)	126 (84.0)	150 (54.9)		
Indian	42 (34.1)	81 (65.9)	123 (45.1)		
^†^ **Marital status**				7.86	0.020 *
Single	11 (13.8)	69 (86.3)	80 (29.3)		
Married	48 (30.0)	112 (70.0)	160 (58.6)		
Divorced/Widowed	7 (21.2)	26 (78.8)	33 (12.1)		
^†,^^⫮^ **Education**				0.760	0.684
Primary education	11 (26.8)	30 (73.2)	41 (15.0)		
Secondary education	37 (25.3)	109 (74.7)	146 (53.5)		
Tertiary education	18 (20.9)	68 (79.1)	86 (31.5)		
^†^ **Total household income**				0.80	0.670
<RM 2300	23 (25.0)	69 (75.0)	92 (33.7)		
RM 2300–5599	26 (26.3)	73 (73.7)	99 (36.3)		
≥RM 5600	17 (20.7)	65 (79.3)	82 (30.0)		
Non-smoker	62 (23.8)	198 (76.2)	260 (95.2)		
Past smoker	4 (30.8)	9 (69.2)	13 (4.8)		
^†^ **Alcohol consumption**				0.03	0.869
Yes	5 (22.7)	17 (77.3)	22 (8.1)		
No	61 (24.3)	190 (75.7)	251 (91.9)		
^†^ **Physical activity**				2.34	0.311
Low	26 (20.6)	100 (79.4)	126 (46.2)		
Moderate	24 (25.0)	72 (75.0)	96 (35.1)		
High	16 (31.4)	35 (68.6)	51 (18.7)		
^†,^^⧧^ **BMI category**					
Mean ± SD	27.3 ± 3.7	22.6 ± 3.6	23.7 ± 4.1	−9.08	0.0001 *
Underweight/Normal weight	18 (10.2)	158 (89.8)	176 (64.5)	52.57	0.0001 *
Overweight/Obesity	48 (49.5)	49 (50.5)	97 (35.5)		

Note: MetS, metabolic syndrome; BMI, body mass index; RM, *Ringgit Malaysia*; SD, standard deviation; IQR, interquartile range. ^†^ Variables are presented as n (%), and tested with Chi-square test with value reported in $\chi^{2}$ and *p*. ^⧧^ Variables are presented as Mean ± SD, and tested with Independent samples *t*-test with value reported in *t* and *p.* ^⏀^ Variable is presented as *n* (%), and reported with Fisher Exact test as more than 20% of the cells had expected count of less than 5. ^⫮^ Education level was merged into three categories to perform valid Chi-square test with value reported in $\chi^{2}$ and *p.* * Indicates a significant difference at *p* < 0.05 by Chi-square test, Fisher Exact or Independent samples *t*-test.

**Table 4:** Logistic regression analysis of MetS risk factors among vegetarians.

Variables	Univariate Logistic Regression		Multiple Logistic Regression			
			^⏀^ Model 1		^∓^ Model 2	
	Crude OR (95% CI)	*p* Value	Adjusted OR (95% CI)	*p* Value	Adjusted OR (95% CI)	*p* Value
**Vegetarian Classifications**						
Lacto-ovo-vegetarians	1.00	1.00	-	-	-	-
Ovo-vegetarians	0.65 (0.14–3.01)	0.586	-	-	-	-
Lacto-vegetarians	2.04 (1.07–3.86)	0.030	-	-	-	-
Vegans	1.41 (0.65–3.05)	0.389	-	-	-	-
**Years of practising vegetarianism**	1.05 (1.02–1.08)	0.001	1.03 (1.00–1.07)	0.039	1.03 (1.00–1.06)	0.096
**Reasons for adopting vegetarianism**						
**Animal welfare**						
Yes	1.00	1.00	-	-	-	-
No	1.52 (0.85–2.74)	0.160	-	-	-	-
**Health concern**						
Yes	1.00	1.00	-	-	-	-
No	1.16 (0.66–2.01)	0.608	-	-	-	-
**Religious belief**						
Yes	1.00	1.00	-	-	-	-
No	0.71 (0.36–1.41)	0.331	-	-	-	-
**Environmentally friendly**						
Yes	1.00	1.00	-	-	-	-
No	1.87 (1.05–3.34)	0.034	-	-	-	-
**Family influence**						
Yes	1.00	1.00	-	-	-	-
No	1.16 (0.48–2.81)	0.746	-	-	-	-
**Others**						
Yes	–	–	-	-	-	-
No	–	–	-	-	-	-
**Age**	1.03 (1.01–1.05)	0.013	-	-	-	-
**Sex**						
Male	1.51 (0.85–2.66)	0.157	-	-	-	-
Female	1.00	1.00	-	-	-	-
**Ethnicity**						
Chinese	1.00	1.00	-	-	-	-
Indian	2.72 (1.53–4.83)	0.001	-	-	-	-
**Marital status**						
Single	1.00	1.00	-	-	-	-
Married	2.69 (1.31–5.53)	0.007	-	-	-	-
Divorced/Widowed	1.69 (0.59–4.82)	0.328	-	-	-	-
**Education levels**						
Primary education	1.39 (0.58–3.29)	0.460	-	-	-	-
Secondary education	1.28 (0.68–2.43)	0.446	-	-	-	-
Tertiary education	1.00	1.00	-	-	-	-
**Total household income**						
Low	1.28 (0.63–2.60)	0.505	-	-	-	-
Middle	1.36 (0.68–2.73)	0.385	-	-	-	-
High	1.00	1.00	-	-	-	-
^⧧^ **Smoking behaviour**						
Non-smoker	–	–	-	-	-	-
Past smoker	–	–	-	-	-	-
**Alcohol consumption**						
No	1.00	1.00	-	-	-	-
Yes	–	–	-	-	-	-
**Physical activity levels**						
Low	0.57 (0.27–1.18)	0.131	-	-	-	-
Moderate	0.73 (0.34–1.54)	0.409	-	-	-	-
High	1.00	1.00	-	-	-	-
**BMI categories**						
Underweight/Normal weight	1.00	1.00	1.00	1.00	1.00	1.00
Overweight/Obesity	8.60 (4.58–16.14)	0.0001	7.54 (3.97–14.30)	0.0001 *	7.74 (4.04–14.82)	0.0001 *

Note: MetS, metabolic syndrome; OR, odds ratios; BMI, body mass index. ^⏀^ Model 1 was adjusted for sex. ^∓^ Model 2 was adjusted for sex and age. ^⧧^ Smoking behaviour and others reason for adopting vegetarianism were excluded from the logistic regression model as more than 20% of the cells had expected count of less than 5. * Indicates a significant difference at *p* < 0.05 in multiple logistic regression. Summary: Nagelkerke R Square = 0.299; Model $\chi^{2}\;$ = 60.95, *p* < 0.05.
